# Author Correction: Multivariate information theory uncovers synergistic subsystems of the human cerebral cortex

**DOI:** 10.1038/s42003-023-04984-y

**Published:** 2023-06-07

**Authors:** Thomas F. Varley, Maria Pope, Joshua Faskowitz, Olaf Sporns

**Affiliations:** 1 School of Informatics, Computing & Engineering, Indiana University, Bloomington, IN 47405 USA; 2 Department of Psychological & Brain Sciences, Indiana University, Bloomington, IN 47405 USA; 3 Program in Neuroscience, Indiana University, Bloomington, IN 47405 USA

**Keywords:** Network models, Dynamical systems, Complexity, Emergence

Correction to: *Communications Biology* 10.1038/s42003-023-04843-w, published online 24 April 2023.

The original Article did not state that Thomas F. Varley and Maria Pope contributed equally to the work. This has now been corrected.

