# Supplementary material for: Multivariate information theory uncovers synergistic subsystems of the human cerebral cortex
Source: Commun Biol. 2023 Apr 24;6:451. doi: 10.1038/s42003-023-04843-w (PMC10125999; doi:10.1038/s42003-023-04843-w)
Supplement: Supplementary file 2 — Supplementary Information [file 42003_2023_4843_MOESM2_ESM.pdf]

# Supplementary Material for Multivariate Information Theory Uncovers Synergistic Subsystems of the Human Cerebral Cortex

Thomas F. Varley[1,2*†], Pope Maria[2,3†], Faskowitz Josh[1,3], Olaf Sporns[1,2,3,1]

[1*]Department of Psychological& Brain Sciences, Indiana University, 1101 East 10th Street, Bloomington, 47405, Indiana, United States.
[2]School of Informatics, Computing, and Engineering, Indiana University, 901 E. 10th Street, Bloomington, 47404, Indiana, United States.
[3]Program in Neuroscience, Indiana University, 1101 East 10th Street, Bloomington, 47405, Indiana, United States.
[4]Indiana University Network Sciences Institute, 1015 E 11th St, Bloomington, 47404, Indiana, United States.

*Corresponding author(s). E-mail(s): tvarley@iu.edu;
†These authors contributed equally to this work.

# Supplementary Figures

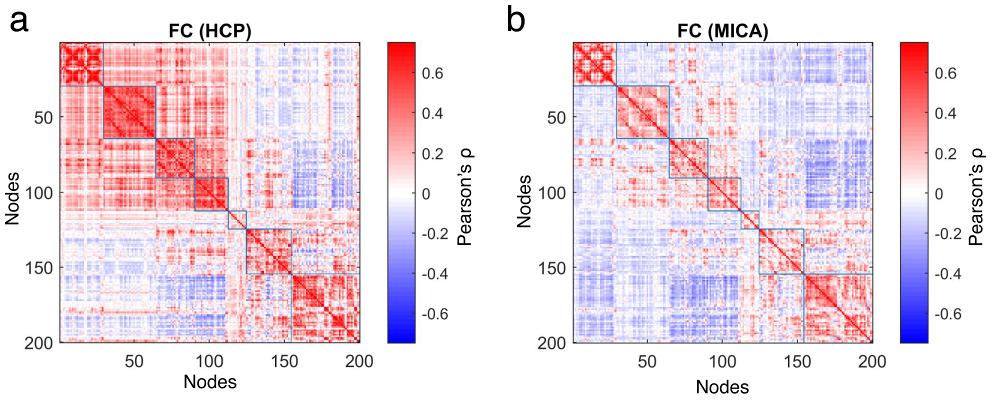


**Supplementary Figure 1 Functional Connectivity (FC) for the two data sets. (a)** Functional connectivity matrix for the HCP data set (95 subjects, 4 runs each). **(b)** Functional connectivity matrix for the MICA data set (50 subjects, 1 run each). The two FC matrices are displayed with canonical functional systems indicated along the main diagonal (top to bottom: VIS, visual; SOM, somatomotor; DAN, dorsal attention; VAN, ventral attention; LIM, limbic; FP, frontoparietal; DMN, default mode). The two data sets are highly correlated ($R = 0.851, p = 0$)

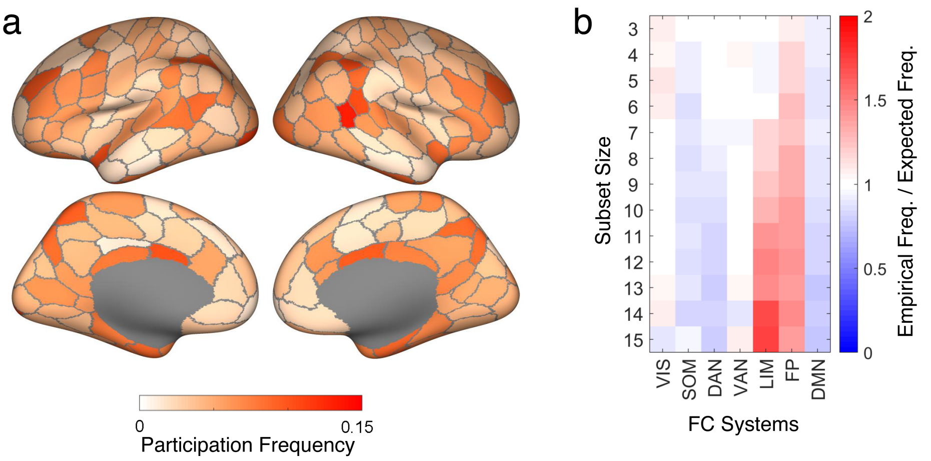


**Supplementary Figure 2 Topography and functional specialization of randomly sampled synergistic subsets in the brain (MICA data).** Compare to Figure4b,c in main text. **(a)** Frequency of individual node participation across 100,000 synergistic 10-node subsets, displayed on a surface rendering of the cerebral cortex indicating the boundaries of the 200 nodes used for constructing the FC matrix. **(b)** Each of the 200 nodes is affiliated with one of 7 canonical functional systems [7]. Frequency of participation of individual nodes in synergistic subsets (negative O-information, subset size ranging from 3 to 15 nodes) is aggregated (averaged) for each functional system. The plot displays the ratio of empirical frequency over the expected frequency if nodes were selected by chance. A ratio $> 1$ or $< 1$ indicates that the system is over-represented or under-represented, respectively, in synergistic subsets.

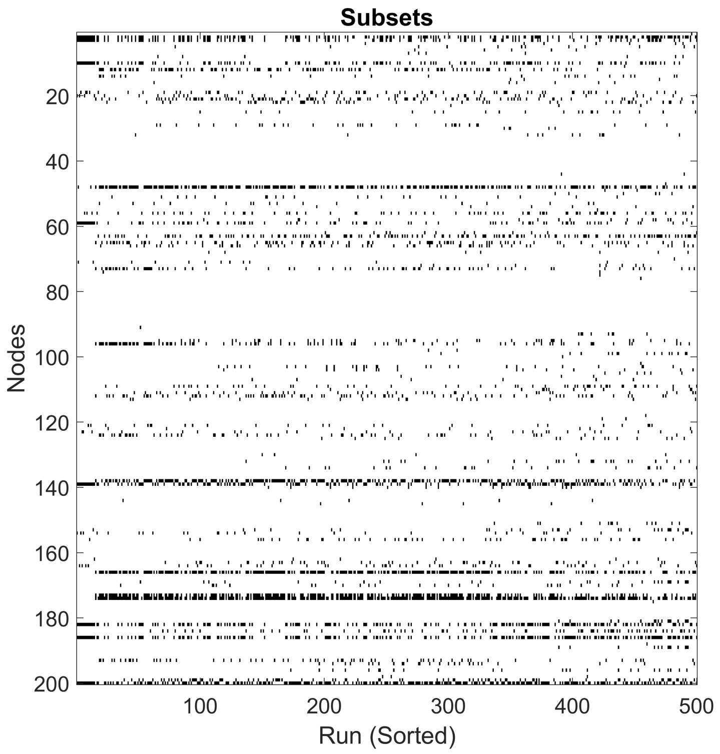


**Supplementary Figure 3 Nodes selected in 500 runs of the optimization algorithm (HCP data, 10-node subsets).** The plot displays the selected nodes (black raster) for each run, sorted by final values of the objective function (here, negative O-information), with the most optimal subsets at the left of the x-axis. Note that multiple runs deliver consistent node configurations.

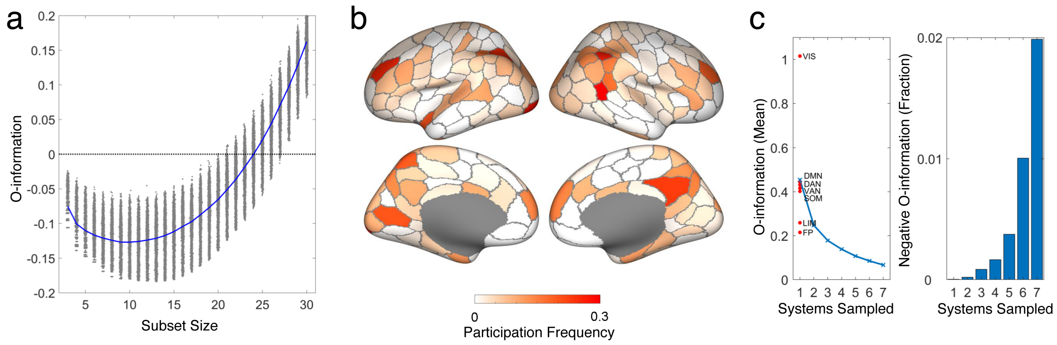


**Supplementary Figure 4 O-Information, brain topography and functional specialization of optimally synergistic subsets identified by simulated annealing** All panels show data from the MICA sample. **(a)** Annealing was carried out 5,000 times for each subset size. plot shows O-information for each optimized subset (gray dots) and their mean (blue line). Note that annealing fails to converge onto any synergistic subsets for subsets containing more than 27 nodes. Optimally negative O-information is achieved for subsets between 8 and 12 nodes, comparable to findings for the HCP data (see main text). **(b)** Frequency of individual node participation across optimally synergistic 10-node subsets (4166 unique subsets out of 5,000 annealing runs), displayed on a surface rendering of the cerebral cortex. **(c)** Mean O-information (left) and fraction of synergistic subsets (right) encountered in samples of 20,000 subsets that contained nodes belonging to between 1 and 7 canonical FC systems (HCP data). The mean O-information for samples obtained exclusively from each of the 7 FC systems is indicated (red dots).

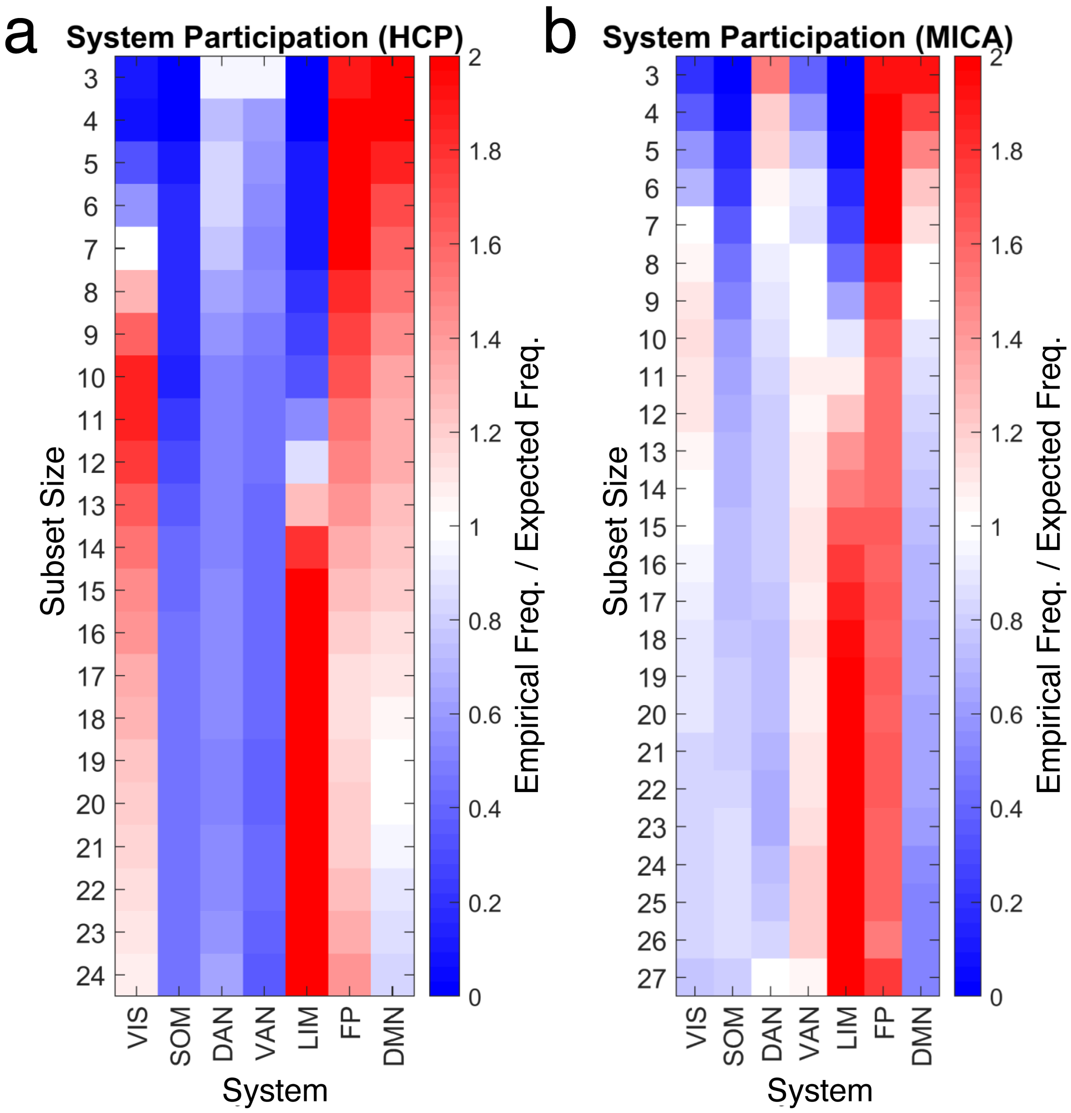


**Supplementary Figure 5 Participation of canonical FC systems in subsets expressing optimal synergy (negative O-information)** Panels **(a)** and **(b)** display data obtained from HCP and MICA FC, respectively. Compare to Figure4 above. Briefly, Each of the 200 nodes is affiliated with one of 7 canonical functional systems [7]. Frequency of participation of individual nodes in synergistic subsets (negative O-information, subset size ranging from 3 to 24/27 nodes) is aggregated (averaged) for each functional system. The plot displays the ratio of empirical frequency over the expected frequency if nodes were selected by chance. A ratio $> 1$ or $< 1$ indicates that the system is over-represented or under-represented, respectively, in synergistic subsets.

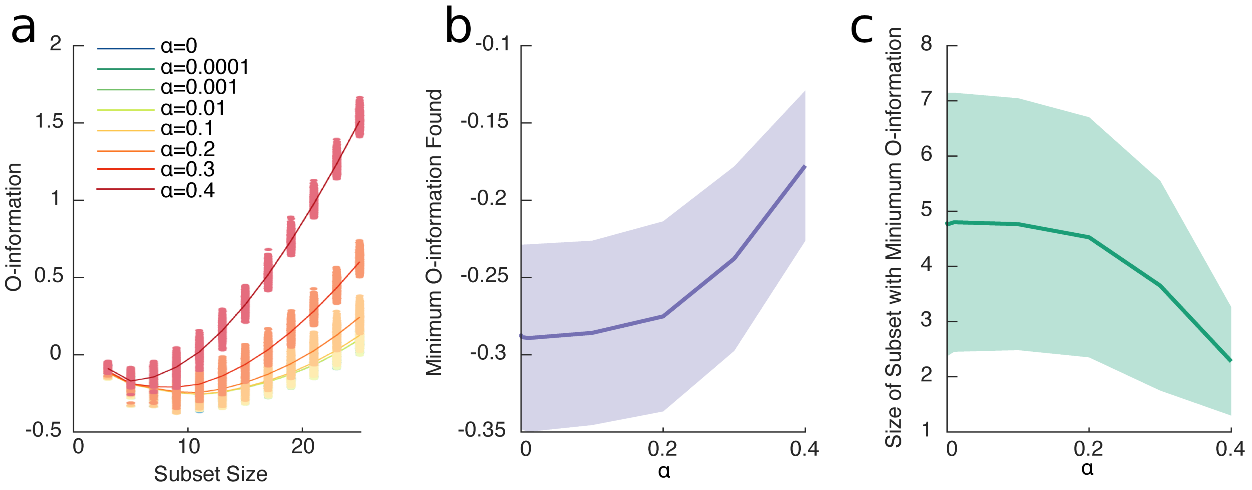


**Supplementary Figure 6 Global signal swamps synergy**. We progressively contaminated the fMRI BOLD data with a globally synchronized sine wave. Each new time series $X'$ was a mixture of the original $X$ and $\sin(t)$. This mixture was controlled by a parameter $\alpha$: $X' = (1-\alpha)X + \alpha\sin(t)$. When $\alpha = 0$, the original data is perfectly preserved, while as $\alpha \to 1$, the data becomes increasingly synchronized. Increasing $\alpha$ can be thought of adding global signal of varying intensities to the data. Importantly, the true synergistic dependencies in the original data are not disrupted by this procedure, only obscured by increasingly strong redundancy. We found that, as $\alpha$ increased, the ability of the simulated annealing optimization to retrieve the optimal subsystems was decreased because the added global signal tipped the redundancy-synergy balance towards redundancy. **A.** This figure shows how the optimal $\Omega$ changes with subsystem size, for various values of $\alpha$. It is apparent that, as $\alpha \to 1$, the optimal value of $\Omega$ increased (became less synergistic). **B.** As $\alpha$ increases, the minimum value of $\Omega$ increased. **C.** As $\alpha$ increases, the size of the optimal ensemble decreases as well. Together, these results suggests that global signal regression (GSR) can be understood as "scrubbing" global redundancies, allowing previously-buried synergies to be seen by the O-information. This may inform when GSR is an essential step in an analysis: if the specific scientific question involves synergies, then GSR may be necessary.

# Supplementary Note 1: Proof that $\mathbf{DTC = N \times C_D}$

Recall the following definitions:

*Description Complexity*

$$C_D(\mathbf{X}) = TC(\mathbf{X}) - \frac{TC(\mathbf{X})}{N} - \frac{\sum_{i=1}^{N} TC(\mathbf{X}^{-i})}{N}$$

*Dual Total Correlation*

$$\begin{aligned} DTC(\mathbf{X}) &= (1-N)H(\mathbf{X}) + \sum_{i=1}^{N} H(\mathbf{X}^{-i}) \\ &= \sum_{i=1}^{N} H(\mathbf{X}^{-i}) - (N-1)H(\mathbf{X}) \end{aligned}$$

Derivation:

$$\begin{aligned} C_D(\mathbf{X}) &= \big[TC(\mathbf{X}) \times \frac{N}{N}\big] - \frac{TC(\mathbf{X})}{N} - \frac{\sum_{i=1}^{N} TC(\mathbf{X}^{-i})}{N} \\ &= \frac{N \times TC(\mathbf{X}) - TC(\mathbf{X})}{N} - \frac{\sum_{i=1}^{N} TC(\mathbf{X}^{-i})}{N} \\ &= \frac{N \times TC(\mathbf{X}) - TC(\mathbf{X}) - \sum_{i=1}^{N} TC(\mathbf{X}^{-i})}{N} \\ &= \frac{(N-1)TC(\mathbf{X}) - \sum_{i=1}^{N} TC(\mathbf{X}^{-i})}{N} \\ &= \frac{(N-1)\big[\sum_{i=1}^{N} H(X_i) - H(\mathbf{X})\big] - \sum_{i=1}^{N} \big[\sum_{i=1}^{N-1} H(X_i) - H(\mathbf{X}^{-i})\big]}{N} \\ &= \frac{(N-1)\sum_{i=1}^{N} H(X_i) - (N-1)H(\mathbf{X}) - (N-1)\sum_{i=1}^{N} H(X_i) + \sum_{i=1}^{N} H(\mathbf{X}^{-i})}{N} \\ &= \frac{-(N-1)H(\mathbf{X}) + \sum_{i=1}^{N} H(\mathbf{X}^{-i})}{N} \\ &= \frac{DTC(\mathbf{X})}{N} \square \end{aligned}$$

# Supplementary Note 2: Higher-Order Information & Entropy Decomposition

To develop a better understanding of higher-order modes of information sharing, we leverage a closely-related area of information theory called *information decomposition* [2**?** ]. Information decomposition was initially proposed as a framework by which the joint mutual information two source variables disclose about a single target ($I(X_1, X_2; Y)$) could be broken down into redundant, unique, and synergistic parts. More recently, attempts have been made to generalize the partial information

decomposition framework to decomposing the joint entropy into different modes of information sharing [4, 5]. While the mathematical details are too complex to fully cover here (interested readers are referred to the citations above), the core of the entropy decomposition rests on defining a notion of "shared" entropy (often called "redundant" entropy in the literature as well). For a set of interacting variables, the shared entropy is the uncertainty that is common to all variables: said otherwise, it is the uncertainty in all variables that can be resolved by observing a single one at random. Following the notation introduced by Ince [4], we refer to this function as the *partial entropy function*. For a set of three interacting variables $\{X_1, X_2, X_3\}$, we refer to the redundant entropy shared by all of them as: $H_\partial(\{1\}\{2\}\{3\})$, where only the indices are included to simplify the notation. Similarly, the information redundantly present in just two variables is denoted as $H_\partial(\{1\}\{2\})$. The partial entropy function has obvious similarities to the definition of Shannon's mutual information (and for Gaussian variables they are often identical [9]). There are key differences however, the most significant being that (as described above) the mutual information is only uniquely defined in the bivariate case and admits multiple multivariate generalizations. Multiple different shared entropy functions have been proposed (for example, Ince proposed one based on the co-information [4], while Finn and Lizier proposed one based on the maximum/minimum local entropy terms [5], and Makkeh et al., proposed a measure based on shared exclusions of probability mass [3]). Conveniently, for our purposes, we do not need to actually define a shared entropy function, it is sufficient to simply assume it exists and proceed with the abstract decomposition.

To build intuition, consider how we might decompose the joint entropy between to variables $X_1$ and $X_2$ into redundant entropy atoms.

$$H(X_1, X_2) = H_\partial(\{1\}\{2\}) + H_\partial(\{1\}) + H_\partial(\{2\}) + H_\partial(\{1, 2\}) \tag{1}$$

The terms can be understood as follows: $H_\partial(\{1\}\{2\})$ is the shared entropy common to both $X_1$ and $X_2$ redundantly. $H_\partial(\{1\})$ is uncertainty intrinsic to $X_1$ that is *not* resolved by observing $X_2$. Finally $H_\partial(\{1, 2\})$ is the "synergistic" entropy: the uncertainty about the joint state of $X_1$ and $X_2$ considered together that is neither shared between $X_1$ and $X_2$ redundantly, nor resolvable by observing either $X_1$ or $X_2$ on its own. By the same logic, the univariate marginal entropies can be decomposed as:

$$\begin{aligned} H(X_1) &= H_\partial(\{1\}\{2\}) + H_\partial(\{1\}) \\ H(X_2) &= H_\partial(\{1\}\{2\}) + H_\partial(\{2\}) \end{aligned} \tag{2}$$

From there we can easily see that:

$$I(X_1; X_2) = H_\partial(\{1\}\{2\}) - H_\partial(\{1, 2\}) \tag{3}$$

This decomposition of mutual information was derived in both the frameworks proposed by Ince [4] and by Finn and Lizier [5] and shows how the mutual information is distinct from the shared entropy: the mutual information is the difference between the shared entropy and the "macro-scale" uncertainty (as an aside, this complicates the usual intuition around mutual information - many users believe they are just

getting $H_\partial(\{1\}\{2\})$ when in fact they are getting $H_\partial(\{1\}\{2\}) - H_\partial(\{1,2\})$, which is compromised by the existence of the higher-order synergy term.

Similar decompositions can be done for the total correlation and dual total correlation, and by extension, the O-information. While it is only practical to do it for the case of $N = 3$ variables (in which case O-information is identical to the co-information [8]), this decomposition provides a complementary perspective to the one introduced above and can help build intuition about what seeing a negative (or positive) O-information tells us about information-sharing in complex systems.

$$
\begin{aligned}
\Omega(X_1; X_2; X_3) = \; & H_\partial(\{1\}\{2\}\{3\}) \\
& -H_\partial(\{1\}\{2,3\}) \\
& -H_\partial(\{2\}\{1,3\}) \\
& -H_\partial(\{3\}\{1,2\}) \\
& -2 \times H_\partial(\{1,2\}\{1,3\}\{2,3\}) \\
& -H_\partial(\{1,2\}\{1,3\}) \\
& -H_\partial(\{2,3\}\{1,3\}) \\
& -H_\partial(\{1,2\}\{2,3\}) \\
& +H_\partial(\{1,2,3\})
\end{aligned} \tag{4}
$$

We can see from equation 4 that $\Omega$ is negative when most of the information is present in greater-than pairwise interactions: for example, $H_\partial(\{1\}\{2,3\})$ refers to information shared between $X_1$ and the joint state of $X_2$ and $X_3$ together (and no simpler combination of sources. Similarly, the exotic term $H_\partial(\{1,2\}\{1,3\}\{2,3\})$, which refers to the information shared by all the pairwise joint states is double-counted. In contrast, $\Omega$ is positive if most of the shared entropy is redundantly present in all three elements ($H_\partial(\{1\}\{2\}\{3\})$). Curiously, the O-information counts the macro-scale synergistic entropy ($H_\partial(\{1,2,3\})$) as positive. The intuitive interpretation of purely synergistic entropy remains uncertain, and so the significance of this second, positively-weighted term in the O-information remains a matter for further research. Notice that the O-information is insensitive to bivariate interactions (e.g. $\{1\}\{2\}$): consequently, for a system composed entirely of bivariate interactions (which is the standard assumption underpinning most statistical network inference pipelines), the O-information will be zero.

How does this decomposition square with the geometric interpretation of redundancy and synergy? If the dominant partial-entropy atom is $H_\partial(\{1\}\{2\}\{3\})$, then the removal of any single element doesn't compromise the overall information structure, since whatever information was lost when that single element was deleted is preserved in the remaining pair. Consequently, the description complexity of $\{X_1, X_2, X_3\}$ is low. Conversely if the dominant partial entropy atom is $H_\partial(\{1,2\}\{1,3\}\{2,3\})$, then the removal of any single element disrupts at least two of the three different collections of elements. That deletion would correspond to a a high description complexity. The PED is explored in more detail in [6].

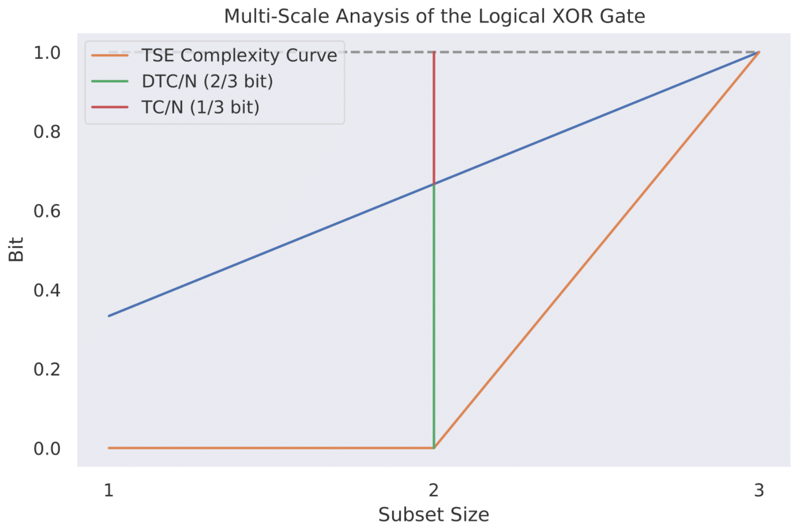


**Supplementary Figure 7 The TSE-complexity curve of the logical XOR function.**

# Supplementary Note 3: Simple XOR Model

This interpretation of negative O-information and synergistic entropy can be made more concrete by considering simple toy systems.

## Logical XOR

The simplest and most well-explored example of synergy in a complex system is that of the logical XOR gate. The gate has a total correlation of 1 bit, and a dual total correlation of 2 bit, leaving an O-information of -1 bit, indicating a synergy-dominated system. The normalized O-information is -1/3, indicating that, on average, the collapse in integration after the removal of any element is greater than what we would expect if information was evenly distributed over all the elements of the system. This can be shown without much difficulty, as the mutual information (or pairwise integration) for any pair of elements $X_1$, $X_2$, and $Y$ is trivially 0. From this insight, we can propose an intuition behind what it means for a variable to be "synergistic" with respect to others: we might say that a variable contributes "synergy" if its presence creates an "information bridge" between variables that, on their own, appear to be uncorrelated. In the XOR example, we know *a priori* that $X_1 \perp X_2$, and so $I(X_1; X_2) = 0$ bit. By the logic of the XOR gate, $I(X_1; Y) = I(X_2; Y) = 0$ bit. By conditioning the bivariate mutual information of any two elements on the third, however, the statistical relationship "becomes visible:" $I(X_1; X_2|Y) = 1$ bit.

# Supplementary References


[1] Williams PL, Beer RD. Nonnegative Decomposition of Multivariate Information. arXiv. 2010 Apr;.

[2] Gutknecht AJ, Wibral M, Makkeh A. Bits and pieces: understanding information decomposition from part-whole relationships and formal logic. Proceedings of the Royal Society A: Mathematical, Physical and Engineering Sciences. 2021 Jul;477:20210110.

[3] Makkeh A, Gutknecht AJ, Wibral M. Introducing a differentiable measure of pointwise shared information. Physical Review E. 2021 Mar;103:032149.

[4] Ince RAA. The Partial Entropy Decomposition: Decomposing multivariate entropy and mutual information via pointwise common surprisal. arXiv. 2017 Feb;.

[5] Finn C, Lizier JT. Generalised Measures of Multivariate Information Content. Entropy. 2020 Feb;22:216.

[6] Varley TF, Pope M, Puxeddu MG, Faskowitz J, Sporns O. Partial entropy decomposition reveals higher-order structures in human brain activity. arXiv; 2023. ArXiv:2301.05307 [cs, math, q-bio]. Available from: http://arxiv.org/abs/2301.05307.

[7] Yeo BT, Krienen FM, Sepulcre J, Sabuncu MR, Lashkari D, Hollinshead M, et al. The organization of the human cerebral cortex estimated by intrinsic functional connectivity. Journal of Neurophysiology. 2011 Sep;106:1125–1165.

[8] Rosas F, Mediano PAM, Gastpar M, Jensen HJ. Quantifying High-order Interdependencies via Multivariate Extensions of the Mutual Information. Physical Review E. 2019 Sep;100:032305.

[9] Barrett AB. Exploration of synergistic and redundant information sharing in static and dynamical Gaussian systems. Physical Review E. 2015 May;91:052802.
