# Supplementary material for: Multivariate information theory uncovers synergistic subsystems of the human cerebral cortex
Source: Commun Biol. 2023 Apr 24;6:451. doi: 10.1038/s42003-023-04843-w (PMC10125999; doi:10.1038/s42003-023-04843-w)
Supplement: Supplementary file 3 — Description of Additional Supplementary Files [file 42003_2023_4843_MOESM3_ESM.pdf]

Description of Additional Supplementary Files

File name: Supplementary Software 1
Description: Contains MATLAB code for computing the O-information, S-information, total correlation, dual total correlation, and TSE-complexity from Gaussian covariance matrices. Also contains code for random sampling and simulated annealing.

File name: Supplementary Data 1
Description: Dataset for reproducing Figure 5.

File name: Supplementary Data 2
Description: Dataset for reproducing Figure 3.
